# Cellular processes associated with LRRK2 function and dysfunction

**DOI:** 10.1111/febs.13305

**Published:** 2015-05-09

**Authors:** Rebecca Wallings, Claudia Manzoni, Rina Bandopadhyay

**Affiliations:** ^1^ Reta Lila Weston Institute of Neurological Studies and Department of Molecular NeuroscienceUCL Institute of NeurologyLondonUK; ^2^ School of PharmacyUniversity of ReadingUK; ^3^ UCL Institute of NeurologyLondonUK; ^4^ Present address: Department of Physiology, Anatomy and Genetics and Oxford Parkinson's Disease CentreUniversity of OxfordOxfordUK

**Keywords:** autophagy, cytoskeleton, genetics, GTPase, kinase, LRRK2, LRRK2 kinase inhibiton, retromer complex, signalling mechanisms, vesicle trafficking

## Abstract

Mutations in the leucine‐rich repeat kinase 2 (LRRK2)‐encoding gene are the most common cause of monogenic Parkinson's disease. The identification of LRRK2 polymorphisms associated with increased risk for sporadic Parkinson's disease, as well as the observation that LRRK2‐Parkinson's disease has a pathological phenotype that is almost indistinguishable from the sporadic form of disease, suggested LRRK2 as the culprit to provide understanding for both familial and sporadic Parkinson's disease cases. LRRK2 is a large protein with both GTPase and kinase functions. Mutations segregating with Parkinson's disease reside within the enzymatic core of LRRK2, suggesting that modification of its activity impacts greatly on disease onset and progression. Although progress has been made since its discovery in 2004, there is still much to be understood regarding LRRK2′s physiological and neurotoxic properties. Unsurprisingly, given the presence of multiple enzymatic domains, LRRK2 has been associated with a diverse set of cellular functions and signalling pathways including mitochondrial function, vesicle trafficking together with endocytosis, retromer complex modulation and autophagy. This review discusses the state of current knowledge on the role of LRRK2 in health and disease with discussion of potential substrates of phosphorylation and functional partners with particular emphasis on signalling mechanisms. In addition, the use of immune cells in LRRK2 research and the role of oxidative stress as a regulator of LRRK2 activity and cellular function are also discussed.

**Abbreviations**
4E‐BP: 4E binding protein
AMK: adenosine monophosphate‐activated protein kinase
COR: C‐terminal of the Roc‐domain
DLP1: dynamin‐like protein 1
EndoA: endophilin A
LRRK2: leucine‐rich repeat kinase 2
LRR: leucine‐rich repeat
MAPK: mitogen‐activated protein kinase
PD: Parkinson's disease
ROC: Ras of complex
ROS: reactive oxygen species
TOR: target of rapamycin
Wnt pathway: Wingless signalling pathway

## Introduction

Parkinson's disease (PD) is an insidious and progressive neurodegenerative disease, affecting around 1–2% of the population over the age of 65 1. The vast majority of PD is sporadic in origin, with only 5–10% being familial 2; because age is the most significant risk factor for the development of the disease, and with an ever‐increasing life span in the western world, disease prevalence is likely to increase. Current treatments ameliorate symptoms but are not capable of slowing disease progression. Moreover, it has been estimated that by the time that motor symptoms emerge, 50–70% of substantia nigra dopaminergic neurons have already degenerated 3; damage which is currently irreversible. It is clear that there is a direct need to increase understanding of PD aetiology and the molecular mechanisms of pathogenesis in order to achieve early diagnosis and identify potential neuroprotective therapies.

Mutations in the gene encoding for leucine‐rich repeat kinase 2 (LRRK2) are the most frequent cause of familial PD 4. LRRK2‐PD has an almost indistinguishable clinico‐pathological phenotype from sporadic PD regarding age of onset, the presence of Lewy bodies (although a minority of cases have been reported without Lewy bodies) 5 and responsiveness to dopamine replacement therapy. These observations, paired with the identification of LRRK2 polymorphisms associated with increased lifetime risk for developing sporadic PD 6, suggested that LRRK2 may provide a deep understanding of the molecular mechanisms of PD. The exact physiological role of LRRK2 is still unknown, although it has been implicated in many cellular functions. In the effort to tackle PD, there is a compelling need for a profound understanding of LRRK2 functions, as well as a description of the signalling pathways in which LRRK2 may be involved in diverse cell types, the identification of regulators of LRRK2 activity, binding partners and phosphorylation targets.

## LRRK2 genetics, protein domain structure; kinase and GTPase activities

In 2004, *LRRK2* was identified as the gene responsible for PD inheritance associated with the *PARK8* locus 7, 8 and was found to be comprised of 51 exons, giving rise to a large (268 kDa) protein. Subsequently, many variants in LRRK2 primary structure have been identified, including dominant mutations segregating with familial PD that also occur in sporadic PD and in cancer 9, together with polymorphisms at the LRRK2 locus that increase the lifetime risk for the development of sporadic PD, but also inflammatory bowel disorder and leprosy 4, 10, 11.

**Figure 1:**
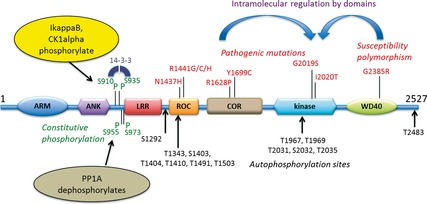
LRRK2 domian structure, pathogenic mutations, constitutive and autophosphorylation sites important for understanding LRRK2 function and dysfunction. Pathogenic mutations and susceptibility polymorphisms are shown in red, constitutive phosphorylation in green and a selection of autophosphorylation sites shown in black. The blue curved arrows depicts intramolecular regulation of kinase activity by other LRRK2 domians; IkappaB and casein kinase 1 alpha (shown in yellow) phosphorylate and PP1 alpha dephosphorylates (shown in fawn) LRRK2 at S910/S935 sites.

LRRK2 is a multidomain protein encompassing two enzymatic functions at its core. The GTPase domain, comprising of Ras of complex protein (ROC) terminating with a spacer domain called the C‐terminal of the Roc‐domain (COR), is immediately followed by the kinase domain, belonging to the serine/threonine kinases. This enzymatic core is surrounded by protein–protein interaction domains comprising the armadillo, ankyrin and leucine‐rich repeat (LRR) domains at the LRRK2 N terminus 12. The LRRK2 C terminus harbours the WD40 domain, which is deemed essential for protein folding, thus controlling LRRK2 function and kinase activity 13 (Fig. 1). Interestingly, the dominant, pathogenic mutations described up to date, occur within the enzymatic core of LRRK2 (Fig. 1), suggesting that modification of LRRK2 activity greatly impacts PD onset and progression. The similarity in PD phenotype and age of onset between homozygous and heterozygous mutation carriers suggests that pathogenic mutations might act by conferring a toxic function on LRRK2 14, 15.

In the overall LRRK2‐PD population, the *G2019S* mutation is the most frequent pathogenic mutation 4. Its occurrence differs among groups; the *G2019S* mutation is rare in Asia, although it is relatively frequent in Southern Europe, reaching a maximum frequency in Ashkenazi Jewish (10–30% of PD patients are *G2019S* carriers) 16 and North African Berber populations (35–40% of PD patients are *G2019S* carriers) 17. The penetrance of the *G2019S*‐LRRK2 mutation appears to have a clear age‐dependent effect and varies from around 50% at age 50, to ~ 74% at age 79 16; although some patients do not manifest any clinical features even in their 80s 18. The *G2019S* mutation occurs in the kinase domain of LRRK2, leading to an increase in kinase activity 19. Cellular toxicity, in both the absence and presence of oxidative stress, and the formation of inclusion bodies were observed when overexpressing *G2019S*‐LRRK2 in cell lines and primary neuronal cultures 20, 21. These results, and the fact that genetic inactivation of LRRK2 kinase activity showed a protective effect against such a toxic phenotype, suggest that an alteration in LRRK2 kinase activity is potentially involved in the neurotoxic and pathogenic mechanisms of LRRK2‐PD. A second mutation in the kinase domain (*I2020T*) was isolated in the Japanese family in which the *PARK8* locus was first described as being associated with PD 22. The effect of this mutation on LRRK2 kinase activity remains controversial 23, 24, 25 and, even with a confirmed increase in kinase activity, it would not be as striking as that reported for *G2019S‐*LRRK2 under the same experimental conditions 26. Nevertheless, *I2020T*‐LRRK2 has been shown to induce toxicity in overexpressing models 19, 27. Combined analysis of LRRK2 toxicity when carrying the *G2019S* or *I2020T* mutation suggests that an increase in LRRK2 kinase activity is sufficient *per se*, but not essential to trigger neurotoxicity.

The kinase function of LRRK2 is of particular interest, especially to pharmacologists, because kinases are typical targets of pharmaceuticals. The LRRK2 kinase domain is thought to assume a typical kinase fold, in which an N‐terminal and a C‐terminal sub‐domain can be identified, with the active site sitting in the cleft between the two. The activation loop, thought to be situated in the C terminus of the kinase domain 28, possesses a DYG motif. In some proteins, this motif undergoes conformational changes that may be related to kinase activity regulation 29. Interestingly, the *G2019S* mutation is situated within this segment of the activation loop and it has been speculated that the glycine residue here imparts conformational flexibility 30; therefore, replacement of the glycine with a serine residue may alter LRRK2 dynamics.

The *R1441C*,* R1441G* and *R1441H* mutations are located in the GTPase domain of LRRK2; the *R1441G* mutation is especially frequent in the Basque population where it accounts for > 40% of familial PD cases 31. Finally, the *Y1699C* mutation lies within the spacer domain, between the GTPase and kinase domains, and is responsible for one of the largest PD pedigrees in the UK with 25 affected subjects over four generations 32. The *R1441C/G/H* mutations showed decreased GTP hydrolysis 33, 34, as did the *Y1699C* mutation 35. Their impact on the kinase function of LRRK2 is still debatable 26; even if an increase in kinase activity is present, it is recorded as a moderate effect in comparison with *G2019S*‐LRRK2. *R1441C*,* R1441G and Y1699C* LRRK2 have been associated with cellular toxicity 27, 36 thus reinforcing the idea that kinase activity is not the only culprit for LRRK2‐induced neurotoxicity. Collectively, this suggests that the pathobiology of LRRK2 is likely to involve the entire enzymatic core, its activity, its folding and potentially its interactions with functional partners.

The presence of a double enzymatic core within the LRRK2 protein suggests that these two functions might influence each other's activities. Autophosphorylation of specific residues (Fig. 1) within the ROC domain has been found to modulate GTP binding 37 and it has been suggested that LRRK2 GTPase activity is regulated by its kinase activity. However, this should be viewed with some caution; autophosphorylation has been observed predominantly in *in vitro* assays as opposed to cellular systems. Moreover, if GTPase activity were regulated solely by autophosphorylation, it would be logical to assume that mutations within the kinase domain would subsequently alter GTP binding/hydrolysis. The hydrolysis of GTP to GDP has been shown to be altered in cell cultures expressing mutations in the GTPase domain 33, 35, 38. However, mutations such as *G2019S*, which occur in the kinase domain of the protein, do not disrupt this 39. Recently, LRRK2 kinase activity was shown to be dependent on GTP binding to the ROC domain 40. In addition, ARHGEF7, the rho guanine nucleotide exchange factor, was identified as an interactor of LRRK2 that could influence GTP hydrolysis activity 41, whereas the guanine exchange nuclear factor GAP (ArfGAP1) markedly reduced GTP hydrolysis and promoted the kinase activity of LRRK2 *in vitro* 42. Furthermore, using a systems biology approach, Dusonchet *et al*. 43 identified regulator of G‐protein signalling 2 (RGS2) as an interactor able to regulate LRRK2 kinase and GTPase activities *in vitro* in a synergistic manner. Clearly, the enzyme activities of LRRK2 undergo intramolecular regulations that can also be influenced by other LRRK2 interactors.

In addition to the known pathogenic mutations, there are a number of coding variants within the *LRRK2* gene which are very rare, and some of these are also present in controls (see Paisan‐Ruiz *et al*. 18 for a detailed review on LRRK2 genetics). Among these, the coding variants *G2385R* in the WD40 domain and *R1628P* in the COR domain act as common PD risk factors among Asian populations 44, 45, 46. The *G2385R* variant essentially doubles the lifetime risk of getting PD 47. It has been shown that the C‐terminally truncated constructs that include the WD40 domain impacts on LRRK2 kinase activity, and a *G2385R* substitution resulted in ~ 50% loss of kinase activity 13. Interestingly, a combined *G2019S*/*G2385R* construct harboured kinase activity similar to WT‐LRRK2 protein, suggesting that these mutations were in fact opposing each other's functions 13. This is further suggestive of a complex interplay of intramolecular interactions within the LRRK2 molecule that is likely to reflect on cellular functions of LRRK2. The rare *N1437H* polymorphism has been found in Scandinavian families 48, 49, but the lack of detailed genetic data makes the pathogenic prediction uncertain. However, overexpression of the construct in HEK293 cells led to increased phosphorylation at Ser1292 50. The genome‐wide association study by Ross *et al*. 47 showed the *LRRK2* locus to be an independent risk factor for sporadic PD. This important finding, together with a similar phenotypic spectrum of LRRK2 patients compared with sporadic cases 51, has fuelled the hypothesis that LRRK2 might also play a role in the pathogenesis of sporadic PD.

One final remark should be on LRRK2′s closest paralogue LRRK1. Despite rare variants in LRRK1 having been proposed to segregate with PD, there is no genetic support for the causal involvement of LRRK1 in disease 52. LRRK1 displays a similar domain organization as LRRK2 53, however, it has been observed that they have specific and independent interactors and are implicated in unique cellular pathways 54. Unfortunately, because of its nonpathogenic relevance, LRRK1 is not a subject of intense study like LRRK2. There are also limited tools developed to study LRRK1; antibodies are not as reliable as those validated for LRRK2, LRRK1 knockout mice 55 and double LRRK2/LRRK1 knockout mice are available (Jackson laboratories) but they have not been extensively studied and there are currently no LRRK1 kinase inhibitors. However, given the close similarity between LRRK1 and LRRK2 and the apparent absence of involvement of LRRK1 in PD, it would be extremely interesting to analyse functional differences between the two enzymes.

## Importance of LRRK2 autophosphorylation and constitutive phosphorylation

Research efforts focussed towards finding molecular substrates of LRRK2 phosphorylation have proven difficult and at the moment, the only recognized target for LRRK2 kinase activity is LRRK2 itself. LRRK2 has been found to autophosphorylate > 20 serine and threonine residues *in vitro* 37, 50, 56, 57, 58, 59, 60, 61. The majority of the autophosphorylation sites reside in the ROC domain, with only a few in the COR and kinase domains (Fig. 1). The physiological relevance of these phosphorylation sites is still not clear; some of them have failed to be detected *in vivo*. In the cellular context, autophosphorylation was observed at T1410 61 and at Ser1292 and this was proposed as a potential measure of LRRK2 kinase activity 50.

Immediately prior to the LRRK2 ROC domain, there is a cluster of serine residues – Ser910, Ser935, Ser955 and Ser973 60, 62, 63, 64 (Fig. 1), which are constitutively phosphorylated; however, they are not autophosphorylation sites. Phosphorylation of these residues is affected by LRRK2 mutations in the ROC/COR/kinase domains, by LRRK2 kinase inhibition and also by extrinsic stressors 62, 65, 66; they are, therefore, used as indirect measures of LRRK2 kinase activity. Dephosphorylation of Ser910/Ser935 affects 14‐3‐3 binding and impacts on downstream signalling 65, 66. The Ikappa B family of kinases has been shown to phosphorylate LRRK2 at Ser910/Ser935 67 and more recently, casein kinase 1 alpha was proposed as the kinase that phosphorylates LRRK2 at these sites 68. However, Protein phosphatase 1A (PPIA) dephosphorylated LRRK2 at Ser910/Ser935 which was reversed using calyculin A 69, a PP1A inhibitor. Further confirmation of PP1A as the phosphatase for LRRK2 constitutive phosphorylation was shown when calyculin A prevented dephosphorylation of LRRK2 at Ser910/Ser935 as a consequence of arsenite‐induced oxidative stress 66. Finding kinases and phosphatases modulating LRRK2 function in a variety of brain cell types and under different cellular contexts will be an important avenue of LRRK2 research that should aid in our understanding of the pathobiology of LRRK2.

## Signalling pathways through LRRK2

**Figure 2:**
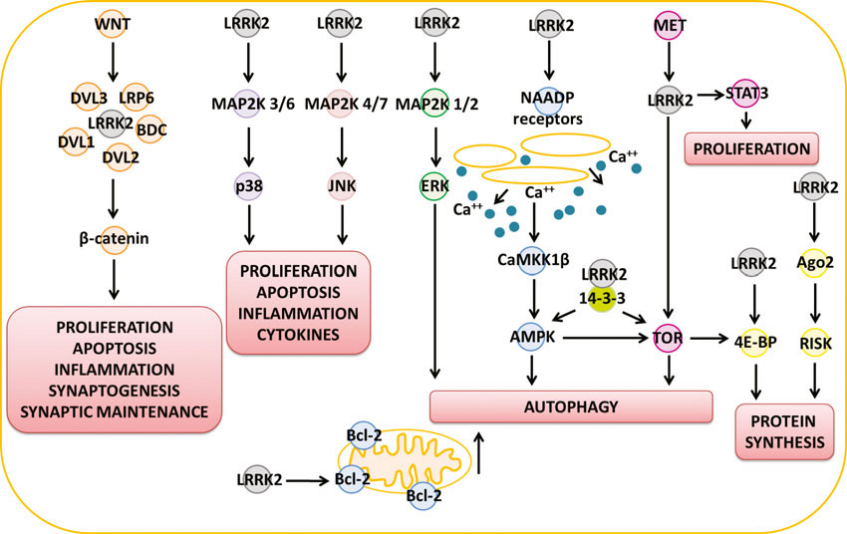
Implication of LRRK2 in signalling pathways. The cartoon depicts multiple signalling pathways that have been associated with LRRK2 function in physiology and/or disease. Signalling pathways are sometimes interconnected and they coordinate the control over multiple cellular activities as reported in the red boxes.

The presence of active kinase and GTPase domains surrounded by protein–protein interaction motifs, suggested analysing LRRK2 in the context of signalling pathways. Indeed, over the past decade, many different signal transduction cascades have been associated with LRRK2 (Fig. 2).

The mitogen‐activated protein kinase pathways (MAPK) were among the first to be investigated as potentially related to LRRK2. MAPK pathways are composed of three layers of proteins able to activate each other in a cascade. At the top layer is a kinase identified as MAP3K that is able to phosphorylate and activate a MAP2K in the second layer, which consequently activates the final MAPK, thus inducing a change in transcription. LRRK2 was able to bind to and phosphorylate MAP2K, ‐3, ‐4, ‐6 and potentially ‐7 *in vitro* 70, 71. Furthermore, a *G2019S*‐LRRK2 transgenic mouse model showed degeneration of dopaminergic neurons in the substantia nigra concomitant with hyperphosphorylation of MAP2K4 72. Activation of MAP2K3 and MAP2K6 is stimulated by stress, cytokines and growth factors, leading to the activation of the downstream effector p38. Activation of MAP2K4 and MAP2K7 is equally sensitive to stress, cytokines and growth factors, leading to the activation of the downstream effector JNK. JNK, and p38, are known to control cell proliferation and differentiation, apoptosis, inflammation, immune responses and the production of cytokines 73.

Another MAPK pathway was recently associated with LRRK2 after MAP2K1 and MAP2K2 (also known as MEK1 and MEK2) were found to be activated by *G2019S*‐LRRK2, leading to hyperphosphorylation of their effectors; ERK1 and ERK2. The alteration of this MAPK pathway was considered responsible for the *G2019S*‐LRRK2 mediated increase in basal autophagy 74.

The Wingless signalling pathway (Wnt) is responsible for the activation of the transcription factor β‐catenin, and is able to regulate nearly 400 genes 73 involved in cell growth, apoptosis, immune functions and inflammation, synaptogenesis during embryonic development and synaptic maintenance in adulthood; alterations in the Wnt pathway have been linked to a loss of synapses during Alzheimer's disease 75. First, the ROC–COR domain of LRRK2 was shown to interact with Dishevelled proteins 1, ‐2 and ‐3 (DVL1–3), key components of the Wnt pathway, whereas LRRK2 pathogenic mutations were sufficient to alter this binding 76. This association has been further characterized and LRRK2 is now hypothesized to have a role interacting with DVL1–3 and other proteins in the canonical Wnt cascade (LRP6 and BDC), thus enhancing the transduction of the Wnt signal 77.

It is interesting to note that LRRK2 is also involved in cancer signalling pathways. Thus, LRRK2 has been associated with MET signalling pathways in papillary renal and thyroid carcinomas; in particular, LRRK2 knockdown was shown to reduce tumour proliferation with concomitant increase in cell death, and reduction of MET signalling through the effectors target of rapamycin (TOR) and signal transducer and activator of transcription 3 (STAT3) 78. In this respect, MET is an oncogene tyrosine kinase receptor that controls multiple pathways involved in cell proliferation and actin organization. MET works by stimulating a variety of downstream effectors, among which are β‐catenin and ERK, thus linking MET with the Wnt and MAPK pathways 79.

LRRK2 has been suggested to modulate nicotinic adenine acid dinucleotide phosphate receptors, with consequent activation of a calcium signalling cascade (mediated by CaMKKβ) that would eventually activate adenosine monophosphate‐activated protein kinase (AMPK) to coordinate different cellular functions related to nutrient homeostasis and energetic balance 80. The CaMKKβ/AMPK pathway for induction of autophagy is sensitive to inhibition by endoplasmic reticulum‐located Bcl‐2 81; intriguingly, it has recently been proposed that *G2019S*‐LRRK2 may be able to bind to and phosphorylate Bcl‐2, with a consequent reduction of the mitochondrial membrane potential, thus stimulating mitophagy 82.

Human LRRK2 and its *Drosophila* orthologue were shown to be able to phosphorylate eukaryotic initiation transcription factor 4E binding protein (4E‐BP) 83. 4E‐BP is a downstream effector in the TOR pathway and its dephosphorylation during nutrient deprivation reduces protein synthesis. However, these data was not reproduced in mammalian cells 84. LRRK2 control over phosphorylation of 4E‐BP was not supported by analysis of sporadic or *G2019S*‐LRRK2 PD brains, or in LRRK2 knockdown or *G2019S* overexpressing mice 85. It is, therefore, possible to argue that results from *in vitro* assays and low‐complex model organisms may not be directly transferable to mammalian systems. However, there are other plausible hypotheses to justify this discrepancy; there may be specific signals needed to activate the function of LRRK2 over 4E‐BP in mammalian systems, or the involvement of LRRK2 within this branch of the TOR pathway may be cell specific, meaning that a cautious selection of cell type is needed to be able to detect it.

Knockin transgenic mice expressing *G2019S*‐LRRK2, as well as LRRK2 knockout mice, exhibited increased expression of mTOR in the kidneys, whereas knockin transgenic mice expressing a kinase inactive form of LRRK2 showed the opposite 86. In the same publication, changes were also described, in the kidneys, for 4E‐BP1 and for protein kinase B (Akt), another hub protein that shares functions with mTOR in the control of the cell metabolism. However, the tissue specificity of these alterations and the complexity of results coming from different genotypes, make the physiological relevance of these results difficult to contextualize without further investigations.

Another study in *Drosophila* proposed that LRRK2 associates with *Drosophila* Argonaute‐1 (dAgo1) and human Argonaute‐2 (hAgo2), thus modulating the RNA‐induced silencing complex 87. A recent finding suggested a component of the protein synthesis pathway, the ribosomal small subunit s15, to be phosphorylated by LRRK2 and sustain cell toxicity in both *Drosophila* model and human neurons 88. Interestingly, the same study reported that s15 was hyperphosphorylated in ribosomal fractions from a small number of G2019S brains compared with controls 88, although this would need further verification using a larger cohort. Another study performed by microarray‐based protein interaction technology and affinity purification coupled by tandem mass spectrometry isolated, among many other positive hits, few ribosomal proteins as possible LRRK2 interaction partners 54, even though they were not selected to be carried on to validation. Similarly, previous immunoprecipitation tandem mass spectrometry isolated, but did not validate, translation initiation factor 2C1 and 2C2 as possible LRRK2 interactors 89. Overall, these reports suggest a possible control of LRRK2 over protein synthesis; but nonetheless, these will need further functional confirmation in mammalian systems for a true physiological function.

A recent study in LRRK2 knockout mice showed that LRRK2 interacts with the PKARIIβ subunit of the protein kinase A holoenzyme regulating its localization. Aberrant localization of protein kinase A in knockout mice increased cofilin and glutamate receptor 1 (GluR1) phosphorylation, thus interfering with synaptogenesis and dopamine signalling through the dopamine receptor Drd1 90.

A final remark should be for the 14‐3‐3 proteins that have been demonstrated to be LRRK2 interaction partners involved in the regulation of LRRK2 cellular localization 62. 14‐3‐3 proteins are able to interact with a plethora of target proteins, thus supporting the function of many different signalling cascades; amongst which are the AMPK 91 and the TOR 92 pathways.

An interesting variety of signalling pathways have been associated with LRRK2 thus far from various reports (see Fig. 2), however, these data have to be considered cautiously because there is still incomplete agreement regarding their relevance. Reproducibility problems arise by the use of different cellular models, different species and sometimes *in vitro* kinase assays that have been difficult to replicate under expression of physiological levels of LRRK2. However, the intriguing idea behind this plethora of possible LRRK2‐modulated signalling pathways is that LRRK2 might play more than one role depending on the cell type in which it is expressed. Moreover, the presence of mutations in the LRRK2 sequence may cause a gain of novel functions, thus implicating LRRK2 in pathology pathways that may not be relevant under physiological conditions. This may support the existence of different functions for LRRK2 in PD, cancer and immune disorders, and it suggests caution in extrapolating general information from different model systems and from experiments involving the use of mutant LRRK2.

## LRRK2 and cytoskeleton

Abnormalities in neurite outgrowth and branching were among the earliest observed LRRK2 cellular phenotypes 93, 94, 95. It was initially proposed that the source of such morphological changes could be a consequence of apoptotic processes 94; however, further studies provided evidence for an association of LRRK2 with tubulin/actin, thus suggesting that such morphological changes may be consequences of LRRK2‐modulation of cytoskeletal dynamics. The GTPase domain of LRRK2 was shown to pull‐down α/β tubulin from cell lysates 96; LRRK2 was coprecipitated with β tubulin from wild‐type mouse brain and recombinant LRRK2 has been proposed to phosphorylate β tubulin *in vitro* 97; a high‐throughput screening to decipher LRRK2 interactome revealed proteins of the actin family and from the actin‐regulatory network to be LRRK2 interactors with LRRK2 able to affect actin polymerization *in vitro* 98, and finally, LRRK2 carrying pathogenic mutations was found to decorate microtubules in cell models 99.

Further work has demonstrated that the interaction between LRRK2 and the cytoskeleton components is not just for the purpose of localization; LRRK2 was found to be able to modulate cytoskeletal dynamics. Disassembly of actin filaments in a process mediated by the GTPase Rac1, was observed in cell lines after LRRK2 knockdown or expression of mutant LRRK2 100. In neuronal cells from *R1441G*‐LRRK2 transgenic mice, as well as in *G2019S*‐LRRK2 fibroblasts, LRRK2 sensitized the actin cytoskeleton to depolymerizing agents 101. LRRK2 was shown to be able to phosphorylate moesin 25, a member of the ezrin/radixin/moesin (ERM) protein family involved in regulation of actin and microtubule structure; *G2019S*‐LRRK2 transgenic and LRRK2 knockout mice were shown to have alterations in the pool of filamentous actin in the filopodia as a consequence of alterations of ERM proteins phosphorylation 95.

*R1441C* and *Y1699C*‐LRRK2, but not *G2019S* or wild‐type LRRK2, were found to decorate nonacetylated microtubules in cell lines and to alter axonal transport in rat neuronal cultures and in *Drosophila* with a mechanism dependent on microtubules acetylation 102. LRRK2 binding to tubulin was associated with modulation of microtubule stability and acetylation 103. The stabilization of microtubules by LRRK2 may be mediated by LRRK2′s interaction with microtubule‐associated protein tau, because it has been demonstrated that LRRK2 is capable of phosphorylating tau in the presence of tubulin, thus altering microtubule–tau binding dynamics 104. Furthermore, introduction of human LRRK2 into a mouse model of tauopathy increased tau phosphorylation at various epitopes and changed its aggregation properties 105.

It is difficult to find a reoccurring theme with LRRK2 findings, and make unique sense of the results obtained in experiments performed with wild‐type LRRK2, mutated forms of LRRK2 and LRRK2 knockout. It is, therefore, difficult to determine whether the regulation of cytoskeleton dynamics is a normal LRRK2 physiological feature, if it is altered during disease, thus contributing to pathogenesis, or if it is a function that gains relevance during disease only. Moreover, it is still not clear whether mutations in the GTPase or kinase domain of LRRK2 affect the regulation of cytoskeleton dynamics to the same extent.

However, a putative function of LRRK2 in cytoskeletal dynamics is intriguing, not only because it could elegantly recapitulate morphological alterations observed in cellular models of LRRK2, but also because it lends to the possibility that LRRK2 may be involved in development and even govern different functions in development and adult life.

## LRRK2 and autophagy

Autophagy was initially associated with LRRK2 when blocking macroautophagy through the knockdown of essential autophagy proteins (Atg7 and Atg8) was sufficient to attenuate the toxicity of overexpressed *G2019S*‐LRRK2 in SHSY5Y cells 106. A following report localized LRRK2 to autophagic vesicles and multivesicular bodies, whereas the knockdown of endogenous LRRK2 was found to be sufficient to induce macroautophagy in HEK293 cells 107. Numerous studies have followed this route of investigation, analysing the role of LRRK2 in autophagy. However, different approaches and model systems have been used to study LRRK2 and it is still not known whether the role of LRRK2 may be different throughout cell lines. The effect of LRRK2 overexpression may be different with respect to studies at endogenous levels, and it is not known whether the two enzymatic domains within LRRK2 orchestrate facilitating or opposing functions. Thus, although there is evidence implicating LRRK2 in autophagy, the extant literature is not sufficient, and at times controversial, in describing the molecular mechanisms through which this association happens. First, LRRK2 has been found to be a degradation substrate of chaperone‐mediated autophagy. Overexpression of *G2019S* or *WT‐LRRK2* was able to reduce the payload of chaperone‐mediated autophagy, indicating that an accumulation of α‐synuclein, and misfolded proteins in general, as seen in PD, may be a partial consequence of a LRRK2‐mediated alteration of cellular proteolytic pathways 108. LRRK2 kinase inhibitors and knockdown 109, 110, 111, as well as LRRK2 overexpression in cell models 80, were able to modify the macroautophagic flux *in vitro*; however, it is still debatable whether LRRK2 possesses a positive or negative regulatory role in the control of macroautophagy and if the role of LRRK2 resides within the initiation or the clearance steps. This open debate has been further emphasized by the study of LRRK2 knockout animal models. Even though the brain of LRRK2 knockout mice did not recapitulate the pathological hallmarks of PD, a biphasic alteration in macroautophagy has been observed in the kidneys, with enhanced autophagy at young ages and reduced autophagy at old ages 112. The use of human fibroblasts carrying LRRK2 pathogenic mutations has confirmed an alteration in autophagy with reports suggesting an increase in basal macroautophagy in G2019S carriers 113, or an impaired response to starvation‐induced macroautophagy across mutations in the LRRK2 catalytic core (*G2019S*,* Y1699C* and *R1441G*) 114. The use of induced Pluripotent Stem cell (iPSC)‐derived, human dopaminergic neurons carrying *G2019S*‐LRRK2 has confirmed a reduction in macroautophagy in comparison with healthy controls 115; but again, details of the molecular mechanism underlying this are still ambiguous. The recent identification of potential interactors of LRRK2 such as Rab7L1, GAK, BAG5, Rab32 and endophilin A (EndoA) 116, 117, 118, and the description of an autophagy/lysosomal phenotype that can be corrected by Rab9 in mutant *Drosophila* 119 suggest that the study of LRRK2 in autophagy should probably be considered with a much wider prospective, taking into account a possible involvement of LRRK2 in vesicles dynamics in general.

## LRRK2 function in vesicle dynamics and retromer function

Recent accruing evidence suggests a role for LRRK2 in vesicle dynamics and retromer function. LRRK2 was found to be associated with membranous structures and vesicles in the mammalian brain 120 and enriched in the Golgi complex 42, 94 at the extent that mice with wild‐type and *G2019S*‐LRRK2 overexpression presented fragmentation of the Golgi complex 121.

LRRK2 has been described as regulating synaptic endocytosis via association with Rab5b; siRNA knockdown of LRRK2 markedly reduced synaptic vesicle endocytosis 122, which was reversed by the introduction of Rab5B. In mammalian cells, interaction was seen between LRRK2 and the dynamin GTPase superfamily 123 involved in membrane scission during clathrin‐associated endocytosis. In *Drosophila*, LRRK2 was shown to phosphorylate EndoA, decreasing EndoA affinity for membranes and affecting EndoA‐dependent membrane tubulation. The *G2019S* mutation impeded synaptic endocytosis 116 that was restored by pharmacological inhibition of LRRK2 kinase activity in G2019S overexpressing flies. Knockout of EndoA led to neurodegeneration 73, thus linking LRRK2‐associated defects to PD. These results were recently further validated in mammalian cells in which LRRK2 was described as being able to phosphorylate neuronal‐specific EndoA1 124.

LRRK2 has been proposed to participate in the control of synaptic vesicle exocytosis by phosphorylating Snapin, and thus regulating soluble NSF attachment protein receptor (SNARE) complex functionality and late endosomal transport 125. Alterations in the amount of ready releasable vesicles have been described in cell models overexpressing *G2019S‐*LRRK2 126. LRRK2 silencing in primary cortical neurons showed altered vesicle‐recycling dynamics and increased vesicle kinetics, suggesting a role for LRRK2 in the control of vesicle pools within the presynaptic bouton 127. Furthermore, inhibition of LRRK2 kinase activity was proven to reduce neurotransmitter release, thus impacting on presynaptic functionality (103). Studies in LRRK2 knockout rats proposed LRRK2 to take part in the control of vesicles exocytosis in lung cells 128.

Recently, LRRK2 has been described as colocalizing with Sec16A, a protein involved in the formation of the endoplasmic reticulum exit site. Loss of LRRK2 led to a reduction in protein transport to the dendritic spine with a consequent reduction in glutamate receptors onto the synapse surface 129.

Two interesting studies by MacLeod *et al*. 130 and Beilina *et al*. 118 showed a genetic interaction between LRRK2 and Rab7L1; a genetic risk factor for sporadic PD. Expression of *G2019S*‐LRRK2 in primary neurons induced lysosomal swelling and accumulation of a component of the retromer complex; the mannose phosphate receptor 130. The mannose phosphate receptor is normally recycled between endolysosomes and the Golgi apparatus 131. The sorting defect was rescued by the overexpression of the retromer component VPS35, as well as the overexpression of Rab7L1 130. Subsequently, Rab7 was found in complex with LRRK2 to cooperatively promote clearance of Golgi‐derived vesicles through the autophagy–lysosomal system. The pathogenic mutations, *G2019S*,* R1441C* and *Y1699C* enhanced Golgi clearance, but the hypothesis‐testing mutations that decrease GTP binding, the T1348N, or the kinase‐inactive K1906M, did not sustain Golgi clearance, suggesting that both kinase and GTPase activities are required for maintaining this cellular process 118. Overexpression of VPS35 exhibited protective effects in mutant LRRK2 *Drosophila* 132; it is of interest to note that mutations in the VPS35 encoding gene have been identified in PD families 133, 134, further implicating the disruption of retromer mediated protein sorting as potentially leading to PD.

Many LRRK2 transgenic models have been created in an attempt to model PD. Although none have alterations resembling PD and the vast majority show no signs of neurodegeneration either, some exhibit a variety of synaptic alterations 135 such as altered striatal dopamine release and/or uptake 136, impairment of dopamine signalling through D2 receptors 137, impaired dopamine reuptake 138, alteration of glutamatergic transmission 139, impaired synaptic vesicles endocytosis with ultrastructural abnormalities in striatal neurons 124 and decreased extracellular dopamine levels in the presence of unaltered synthesis, storage and uptake 140. These findings may be seen as a confirmation of a putative LRRK2 function at the synapses; however, it is very difficult to harmonize different results coming from different models and combine them to eventually draw an exhaustive picture of the molecular mechanism supported by LRRK2. At the moment, we can only confidently state that the bulk of all these studies suggest that LRRK2 may be associated with a complex array of cellular functions involving vesicle dynamics, trans‐Golgi networks and autophagy/lysosomal homeostasis. The intriguing hypothesis is that the synergism of all these membrane dynamics may be controlled by LRRK2 and may be at the molecular base of PD neurodegeneration.

## LRRK2, reactive oxygen species and mitochondria

LRRK2′s putative association with mitochondria suggests that it might play a role in mitochondrial dysfunction driving PD pathogenesis. Indeed, fibroblasts from PD patients carrying the *G2019S* mutation showed abnormal mitochondrial morphology 141. Similarly, wild‐type LRRK2 overexpression in SH‐SY5Y cells caused mitochondrial fragmentation, which was further exaggerated by the *R1441C* and *G2019S* mutations 142. In *G2019S* transgenic mice, ultrastructure examination showed an accumulation of damaged mitochondria, consistent with altered mitophagy in aged mice 143. In a double‐transgenic mouse expressing *G2019S*‐LRRK2 and *A53T* α‐synuclein, structural and functional abnormalities within the brain mitochondria suggested LRRK2 to induce a mitochondrial phenotype 121. Overexpression of *G2019S*‐LRRK2 in SH‐SY5Y cells caused mitochondrial uncoupling, leading to reduced membrane potential and increased oxygen consumption 144. Primary mouse cortical neurons expressing either *G2019S* or *R1441C‐*LRRK2 demonstrated increased mitophagy associated with altered calcium levels 145. Although human iPSC‐derived neurons carrying *G2019S* or *R1441C*‐LRRK2 showed normal mitochondrial electron transport chain, they showed increased vulnerability to chemical stressors and disrupted mitochondrial movement 146. LRRK2 overexpression caused the recruitment of dynamin‐like protein 1 (DLP1) protein to the mitochondria 142. Similarly, coexpression of DLP1 and LRRK2 induced increased oxidative stress, DLP1 relocation to the mitochondria and promoted mitochondria clearance 147. Finally, inhibition of DRP1 was able to rescue mitochondrial fragmentation in both *G2019S* expressing HEK cells and *G2019S*‐LRRK2 fibroblasts 148. These observations suggest that LRRK2 might be responsible for mitochondria homeostasis, possibly via DLP1‐dependent, mitochondrial quality control. However, it remains to be determined whether this truly acts as a primary pathogenic event in LRRK2‐PD, or if mitochondrial damage happens just as a secondary consequence of LRRK2‐induced toxicity.

The same causative hierarchy is yet to be determined for the association of LRRK2 with reactive oxygen species (ROS). Elevated ROS has been implicated as a pathological feature of PD; *WT‐*LRRK2 may be neuroprotective, attenuating H_2_O_2_‐induced cell‐death in HEK293 and SH‐SY5Y cells 149, whereas iPS cells carrying the *G2019S* mutation were found to be more sensitive to H_2_O_2_ exposure with increased caspase **3** activation and cell death 150. Similarly, mitochondrial dysfunction has been linked to increased ROS production in LRRK2 mutant cells 147.

Several mechanisms have been proposed to control the link between increased vulnerability to ROS and LRRK2 neurotoxicity. For example, increased kinase‐dependent interactions were shown between LRRK2 and two of its hypothetical substrates, DLP1 147 and peroxiredoxin 3 (PRDX3) 151. However, increased vulnerability to ROS was also described after overexpression of mutations outside the kinase domain as well as with kinase dead mutations in *Caenorhabditis elegans* 152, again suggesting that the molecular function of LRRK2 is likely to be governed by a delicate balance between kinase and GTPase activities. More recently, studies from our laboratory have shown that oxidative stress caused by arsenite led to altered biochemical properties of LRRK2 protein. Arsenite‐induced stress caused LRRK2 self‐association, inhibited its kinase activity, abrogated the GTP binding and translocated LRRK2 into centrosomes 66. In the context of exogenous stress and LRRK2 properties, it will be important to study other relevant PD stressors in different cellular phenotypes.

## LRRK2 and the immune system

Despite LRRK2 having ubiquitous expression, substantial levels of LRRK2 protein and mRNA are present in peripheral blood mononuclear cells, lymph nodes, spleen 153 and primary microglia 154. There is no definitive description of LRRK2 function in immune cell lineages and how this may contribute to disease pathogenesis. It has been proposed that within the immune system, LRRK2 may be involved in the activation and maturation of immune cells 155, in controlling the radical burst against pathogens in macrophages 156, and in modulating neuroinflammation through cytokine signalling 157, 158. A more detailed link between LRRK2 and neuroinflammation in PD has been discussed recently by Greggio and colleagues 159.

The manifestation of these very specific functions of LRRK2 within the immune system opens up the intriguing hypothesis that LRRK2 may play different roles within different cell types and tissues. This scenario could be explained only by the presence of molecular mechanisms allowing the same LRRK2 protein to behave differently in different tissues; those mechanisms may be based on cell‐type‐specific LRRK2 differential splicing, or on tissue‐specific expression of LRRK2 activators, substrates and partners. Little is known about LRRK2 splicing and it may be reasonable to suppose that different cell types express different LRRK2 isoforms potentially involved in different cellular functions. The first study performed in mice showed indeed a differential expression of two splicing variants of LRRK2 in primary neurons, astrocytes and microglia 160, but more investigations are required.

**Figure 3:**
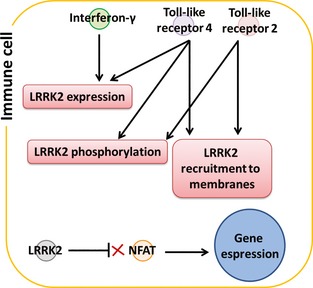
Implication of LRRK2 in immune‐specific functions. The cartoon summarizes the LRRK2‐specific events that have been described to occur in cells from the immune system.

Different cell lineages may express different LRRK2 partners able to specifically regulate LRRK2 levels and phosphorylation; features that are supposed to be linked with LRRK2 activation/repression. This assumption was demonstrated by the observation that LRRK2 expression and activity can be modulated via immune‐cell‐specific signalling pathways (Fig. 3). For example, LRRK2 expression can be induced in peripheral blood mononuclear cells by interferon‐γ 156, 161; or in monocytes after triggering their maturation to dendritic cells and macrophages 155. It was also demonstrated that in a macrophagic cell line (RAW264.7) and a microglial cell line (BV2), stimulation of toll‐like receptors 2 and 4 (TLR2, TLR4) was able to increase phosphorylation of LRRK2 and induce its recruitment to membranes 67, 109. Finally, toll‐like receptor 4 stimulation was able to increase LRRK2 expression and phosphorylation in primary rat microglia 158. LRRK2 was described as a negative regulator of nuclear factor of activated T cells (NFAT), a protein involved in transcriptional regulation in T cells, macrophages, dendritic cells and neutrophils 162; reinforcing the hypothesis that LRRK2 may have a precise role in the immune system because of the peculiar interaction partners through which it can exert tissue‐specific functions. The pathological implications of these observations are intriguing in two ways. First, this enforces the relevance of astrocytes and microglia in PD alongside with neurons. Indeed, activated microglia and monocytes, as well as increased cytokine levels, were reported in PD brains 26. Second, a putative role of LRRK2 in the regulation of the immune response may justify the genetic association of LRRK2 with the susceptibility to inflammatory bowel disorder 10 and leprosy 11 other than with PD.

## LRRK2 kinase inhibition: therapeutic potential

The fact that LRRK2‐PD has an almost indistinguishable pathological phenotype from sporadic PD and the presence of a druggable kinase activity within LRRK2 were sufficient reasons for researchers to look at this protein as a potential target for a neuroprotective treatment in PD. Most notably, the development of selective pharmacological inhibitors of LRRK2 kinase activity is an active area of research with at least six potential LRRK2 inhibitors described in the literature and many leading pharmaceutical companies working on LRRK2 research programmes (see Ref. 163 for a review). Although some of the earlier inhibitors had rather limited use because of their nonselective effects in cells and their inability to cross the blood–brain barrier 164, 165, the development of LRRK2‐IN‐1 166 provided the first step towards a pharmacological tool to define the biological role of LRRK2, at least in cell models. On the flip side, LRRK2‐IN‐1 is not brain penetrant and also has inhibitory effects on ERK5 161, a critical enzyme and therefore ruling out its usefulness in clinical settings. Furthermore, it has recently been demonstrated that LRRK2‐IN‐1 exhibits significant off‐target effects, independently of LRRK2, including the inhibition of tumour necrosis factor alpha in astrocytes and increased neurite branching and length in neurons 167; perceived pathways of LRRK2 pathology. Not only does this highlight the problematic use of LRRK2‐IN‐1 when investigating LRRK2 function, but also negative off‐target effects in a therapeutic context.

Following the initial screening for LRRK2 inhibitor compounds, more brain‐penetrant LRRK2‐specific inhibitors were developed soon thereafter. The compound HG‐10‐102‐01(4) showed selective inhibition of WT and *G2019S*‐LRRK2 at micromolar concentrations in mouse brain and also inhibited Ser910/Ser935 phosphorylation 168. The compound GSK2578215A 169 was highly selective for LRRK2 kinase inhibition when compared with more than 450 other kinases tested. However, both these compounds have limited pharmacokinetic properties that exclude these from testing in clinical trials in humans.

Based on the structure of the HG‐10‐102‐01(4) compound, two further compounds were developed recently; the GNE‐0877 and GNE‐9605 170. Both these compounds demonstrated high selectivity, potency, brain penetrance and good metabolic clearance and stability when tested *in vivo* in rat models expressing human LRRK2 (see Ref. 171 for a review). The improved pharmacokinetic profile supported the hypothesis that these compounds can be safely tested in higher order animals and could potentially be entered into the preliminary stages of drug screening.

Clearly, the developments in identifying LRRK2 kinase inhibitors and a potential to treat PD have now entered an exciting phase but should be viewed with cautious optimism. A recent report demonstrated abnormal lung, kidney and liver pathology in LRRK2 knockout rats compared with rats expressing physiological levels of LRRK2 172. Crohn's *T2397M*‐mutant LRRK2 patients have reportedly lower levels of LRRK2 protein activity within their immune cells 162, suggesting that one harmful side effect of a complete inhibition of LRRK2 as a treatment for PD could be the development of intestinal‐immune diseases. What would be necessary now is the investigation of LRRK2 inhibitors in higher order mammalian models including their long‐term effects on the immune cells and peripheral organs in order to assess safety 173. The efficacy of such a therapy will also need to be demonstrated, and confirmation of the physical substrates of LRRK2 will aid in designing alternative activity assays for such inhibitors. As well, a general consensus of a model for LRRK2‐mediated pathology will need to be agreed upon in order for a validation criteria to be established 173. Importantly, for clinical use, the long‐term benefits of LRRK2 inhibitor treatment should outweigh the advantages of the already existing symptomatic treatments for PD 174. In this endeavour, it will be important to broaden the search for LRRK2 inhibition to include domains outside of the kinase domain 175, 176.

## Concluding remarks

**Figure 4:**
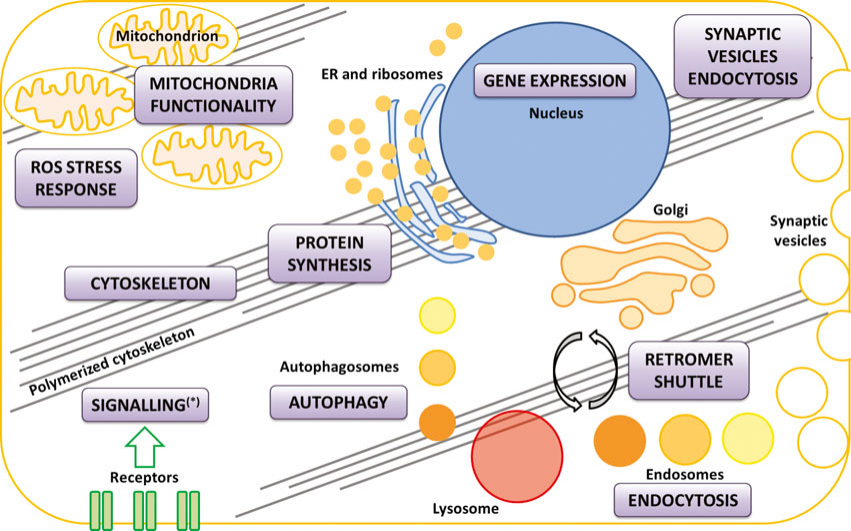
Implication of LRRK2 in cellular functions. The cartoon represents the cellular processes (red boxes) that have been associated with LRRK2 function in physiology and/or disease. The data are from a multitude of experiments run by laboratories across the world over the past decade and are based on different model systems and experimental approaches. It is not possible to describe a hierarchy among these processes, or score them based on reliability. More investigations are needed to determine which of these processes are directly controlled by LRRK2 and which appear to be LRRK2 regulated or whether they are just consequences of other LRRK2 primary functions. It remains to be determined if all of these functions co‐occur in a single cell type or whether LRRK2 orchestrate different, specific functions in different cell types.

It is evident that there is still much to be understood about the LRRK2 protein regarding both its physiological and neurotoxic properties. A large variety of functions have been associated with LRRK2, both in terms of its physiological and cellular roles, and its pathological role during neurodegeneration (Fig. 4). These constitute an intricate network of LRRK2 putative functions making it central to two recent bioinformatics articles that have analysed LRRK2 interactome by means of protein–protein interaction databases and repositories of cellular pathways 177, 178. Instead of a clarifying mechanism of LRRK2‐PD, this wide range of implicated functions makes interpreting LRRK2′s role in disease even more challenging. Many questions are still unanswered. This is, in part, due to the complex protein structural domains of LRRK2, as well as the variety of models used in LRRK2 research. What needs to be resolved now is the ability to distinguish between LRRK2 physiological functions and those that are acquired during pathology, and to discriminate between primary and secondary pathophysiological events.

It is still unknown whether LRRK2 may interpret different roles in different cell types, thus giving origin to cell‐specific phenotypes; in light of this, LRRK2 functions may be tissue specific, thus giving an explanation for the plethora of activities described up to now in diverse model systems (Fig. 4). Analysis of LRRK2 in different cell types is of extreme interest, as is the investigation of different LRRK2 isoforms in different brain regions, as recently shown by Trabzuni *et al*. 179 in control brains. This, together with further studies of LRRK2‐related biology associated with ROS and PD related toxins, phosphatases and kinases that modulate LRRK2 biology, should lead to increased understanding on LRRK2 function and dysfunction.

It will be crucial for future research on LRRK2 to consider the early events in neurodegeneration, as LRRK2 genetic penetrance varies between 30% and 80% depending on age. Although the loss of dopaminergic neurons is a key pathological characteristic of PD, it is preceded by many other dysregulations, which occur prior to the development of motor symptoms. It is the mechanism of LRRK2 associated with these events that needs to be uncovered. For example, LRRK2 has been associated with Wnt signalling pathways 76, which are essential for the acute regulation of synaptic function; a function that is dysregulated in the premotor symptom stages of rodent models of PD 180. From this prospective, the involvement of LRRK2 in Wnt signalling is an exciting possibility that may give a reason for the alteration in gene expression, as well as disruption of vesicle trafficking 181, which are implicated in PD. Another interesting connection is between LRRK2 and the autophagy/lysosomal/Golgi network, because alteration of the proteolytic balance within the cell may be the reason for the build‐up of toxic aggregates of amyloidogenic α‐synuclein, as implicated in PD. Indeed, the majority of LRRK2 mutation cases show an abnormal accumulation of α‐synuclein‐positive Lewy bodies 182, although it is unclear which α‐synuclein species is more relevant in *G2019S* pathology 183. Identifying signalling molecules that regulate the normal and pathophysiological functions of LRRK2 is a critical unmet need for developing novel therapies and the choice of the most relevant disease models will be critical in this endeavour.

Finally, it is important that all progress in LRRK2 research is interpreted carefully. The development of LRRK2 kinase inhibitors gives us cause for optimism for potential treatment for PD, but clearly the effects of inhibiting kinase activity should be gauged in the context of the entire LRRK2 protein.

## Author contributions

Rebecca Wallings and Claudia Manzoni have contributed equally to the review. Rina Bandopadhyay has thoroughly revised and proof read the manuscript. All three authors have contributed to parts of the manuscript.
